# A multi-dimensional characterization of anxiety in monozygotic twin pairs reveals susceptibility loci in humans

**DOI:** 10.1038/s41398-017-0047-9

**Published:** 2017-12-11

**Authors:** Reid S. Alisch, Carol Van Hulle, Pankaj Chopra, Anita Bhattacharyya, Su-Chun Zhang, Richard J. Davidson, Ned H. Kalin, H. Hill Goldsmith

**Affiliations:** 1 Departments of Psychiatry, University of Wisconsin, Madison, WI USA; 2 Neuroscience Training Program, University of Wisconsin, Madison, WI USA; 3 Waisman Center, University of Wisconsin, Madison, WI USA; 4 Department of Human Genetics, Emory University School of Medicine, Atlanta, GA USA; 5 Departments of Neuroscience, University of Wisconsin, Madison, WI USA; 6 Departments of Neurology, University of Wisconsin, Madison, WI USA; 7 Departments of Psychology, University of Wisconsin, Madison, WI USA; 8 Center for Healthy Minds, Madison, WI USA

## Abstract

The etiology of individual differences in human anxiousness is complex and includes contributions from genetic, epigenetic (i.e., DNA methylation) and environmental factors. Past genomic approaches have been limited in their ability to detect human anxiety-related differences in these factors. To overcome these limitations, we employed both a multi-dimensional characterization method, to select monozygotic twin pairs discordant for anxiety, and whole genome DNA methylation sequencing. This approach revealed 230 anxiety-related differentially methylated loci that were annotated to 183 genes, including several known stress-related genes such as *NAV1*, *IGF2*, *GNAS*, and *CRTC1*. As an initial validation of these findings, we tested the significance of an overlap of these data with anxiety-related differentially methylated loci that we previously reported from a key neural circuit of anxiety (i.e., the central nucleus of the amygdala) in young monkeys and found a significant overlap (*P*-value < 0.05) of anxiety-related differentially methylated genes, including *GNAS*, *SYN3*, and *JAG2*. Finally, sequence motif predictions of all the human differentially methylated regions indicated an enrichment of five transcription factor binding motifs, suggesting that DNA methylation may regulate gene expression by mediating transcription factor binding of these transcripts. Together, these data demonstrate environmentally sensitive factors that may underlie the development of human anxiety.

## Introduction

Anxiety is frequently characterized by a negative affective response that is associated with the anticipation of encountering a potential threat^1^. Trait-like anxiety in humans and non-human primates is associated with stable individual differences in hypothalamic–pituitary–adrenal (HPA) axis activation and amygdala function^2,3^. HPA activation results in the release of cortisol, and increased cortisol concentrations in children and adolescents can be linked to inhibited behaviors and anxiety that often persist throughout life^4,5^. Additionally, a loss of the ‘natural’ circadian decline in afternoon/evening cortisol levels has been correlated with shyness and later alterations in behavior, including internalizing problems^6,7^, suggesting that late-in-the day cortisol levels in children and adolescents may be an index of early life and current stress exposure as well as altered behaviors. High afternoon cortisol levels in childhood are also negatively correlated with amygdala–prefrontal cortex connectivity in adolescents and adults, indicating that a disruption in amygdala function is related to trait-like anxiety^8–12^. In fact, anxiety prone individuals show greater amygdala activation during emotion processing tasks, further supporting a central role of the amygdala in processing of fearful stimuli^13–15^. Moreover, lesions in the central nucleus of the amygdala of non-human primates results in decreased adrenocorticortropic hormone (ACTH) concentrations before and after stressful conditions^16^. Finally, higher and prolonged amygdala metabolism following a stressful challenge results in increased anxiety-like behaviors (e.g., freezing) in young rhesus monkeys^17^, suggesting that the *timing* of amygdala activation and deactivation, in both humans and rhesus monkeys, is associated with trait-like anxiety.

Genetic data suggest that common anxiety disorders like generalized and social anxiety disorders are ~20–40% heritable and that environmental factors—potentially including epigenetic modifications—likely account for much of the remaining variability^18^. Studies using adult post-mortem brain tissue support a role for DNA methylation (*i.e*., 5-methylcytosine (5mC)) in the development of anxiety, bipolar disorder, schizophrenia, and major depressive disorder^19–24^. Our recent study in young monkeys, as well as studies in humans, identified differentially methylated genes that are implicated as risk factors for anxiety and depressive disorders^25,26^. Thus, these studies support the hypothesis that DNA methylation may have an important role in the risk to develop trait-like anxiety. However, these studies have relied heavily on the ability to access brain tissue. Focusing studies on anxiety-related DNA methylation profiles in blood has the potential to provide tools that could be clinically utilized to improve diagnostic and treatment strategies.

Twin studies showed that afternoon cortisol levels and amygdala volume are strongly influenced by environmental (i.e., non-genetic) factors^27–29^. In addition, a substantial portion of the individual variability in anxiety level is due to variations in non-genetic factors^30^. The monozygotic (MZ) twin difference design^1^ is an ideal way to probe non-shared environmentally or experientially based relationships among HPA activity and amygdala function. We recently used this design, with a small sample of MZ twins enriched for anxious behaviors, to show that co-twins with higher afternoon cortisol measured at age 8 years experienced protracted amygdala activation (i.e., poorer recovery from negative images) during an fMRI task at age 15, which suggested that non-shared environmental factors that influence cortisol levels also are related to (or set the stage for) later amygdala (dys)function^9^. Therefore, we anticipated finding an environmentally mediated relationship between cortisol levels and anxiety symptoms in a large birth-based sample of MZ twin pairs. Under the assumption that the MZ co-twin with higher childhood afternoon basal cortisol levels and protracted amygdala response would have more adolescent anxiety symptoms than their co-twin with lower cortisol and amygdala activation, we selected three MZ twin pairs from Burghy and colleagues that were maximally discordant on these phenotypes^9^. In these individuals, whole genome DNA methylation levels were profiled to identify anxiety-related DNA methylation in human blood.

## Materials and methods

### Sample

Between 1997 and 2002, families identified from Wisconsin state birth records^31^ were invited to participate in a research panel, with 74% agreeing. At age 7 years (mean age = 7.1 years, s.d. = 0.6) twins were screened via telephone interview with the primary caregiver (>95% mothers) with the goal of mildly enriching the sample for risk for behavior problems (see Supplementary Figure 1). Children who scored at least 1.5 s.d. above and below the mean on at least one of eight parent-reported symptom scales of the Health and Behavior Questionnaire^7^ were preferentially selected (along with their co-twins regardless of standing). This mild selection was intended to introduce more variance in symptoms than an entirely unselected sample would show. Shortly after screening, selected participants (*N* = 702 total pairs, 299 MZ pairs) took part in a multi-informant, multi-method assessment at age 8 years (mean age = 7.5 years, SD = 0.7). Participants were invited to enroll in two follow-up studies at age 13 (mean age = 13.1 years, s.d. = 1.3*, N* = 503 total pairs, 184 MZ pairs) and age 15 (mean age = 14.4 years, s.d. = 1.5, *N* = 581 total pairs, 214 MZ pairs). Informed consent (and parental permission in childhood) was obtained for all assessments, and participants received monetary compensation. University of Wisconsin–Madison Institutional Review Boards approved all procedures. All methods were carried out in accordance with the approved guidelines.

A subset of MZ twins were also selected for a neuroimaging study if one or both twins experienced chronic anxiety (i.e., met diagnostic criteria for at least one internalizing disorder at two assessment occasions based on either parent or youth report); 13.5% of participants met criteria. Pairs were considered discordant if one twin experienced chronic anxiety and the co-twin did not meet criteria for any anxiety disorder at any assessment occasion (5%); pairs were concordant if both co-twins experienced chronic anxiety (6%). The majority of twin pairs were neither concordant nor discordant. Twenty-four pairs participated in the neuroimaging study (mean age = 15.8 years, s.d. = 1.6).

Characteristics based on demographics collected at age 8 years are shown in Supplementary Table 1 for the sample as a whole and the three twin pairs selected for DNA methylation profiling. The sample is largely non-Hispanic, white (93%) and middle class^31^. On average the mother’s education corresponded to some technical or college experience. Median family income falls in the $50,000–$60,000 range.

### Behavioral measures

Trained interviewers administered the Diagnostic Interview Schedule for Children (DISC)^32^ and Diagnostic Predictive Scales^33^ to parents and adolescent offspring. Parents completed the Health and Behavior Questionnaire^7^ (HBQ) at all waves of data collection. Offspring completed the HBQ during both adolescent assessments. We constructed a general anxiety composite consisting of standardized DISC generalized anxiety disorder symptom counts averaged with standardized HBQ over anxiousness scores, and we constructed a social anxiety composite in the same manner. Parent (age 8 years) and offspring (ages 13 and 15 years) scores were kept separate.

Clinicians administered the Schedule for Affective Disorders and Schizophrenia for School-Age Children (K-SADS^34^) to both parents and adolescent offspring during the imaging visit. The K-SADS provides past and current diagnoses and past and current subclinical symptoms.

### Biological measures

At age 8 years, basal cortisol was assayed from saliva samples collected by parents 30 min after waking, in the late afternoon (between 1500 hours and 1700 hours), and 30 min prior to bedtime on three consecutive days. Cortisol was assessed in duplicate with a salivary enzyme immunoassay kit (Salimetrics, State College, PA, USA). Repeat assays were performed on any samples not meeting quality control requirements. Families were assayed across one or two batches. Log-transformed afternoon cortisol values were regressed on time since waking before averaging over the three collection days.

### fMRI task

At a mean age 15 years, structural and functional images were collected on a 3 T MRI scanner (Discovery MR750, General Electric Medical Systems, Milwaukee, WI, USA) with an 8-channel RF head coil array. T1-weighted structural images (1 mm^3^ voxels) were acquired axially with an isotropic 3D Bravo sequence (TE = 3.18 ms, TR = 8.13 ms, TI = 450 ms, flip angle = 12°). T2*-weighted gradient-echo echo-planar pulse sequence images were collected during with TE = 25 ms, TR = 2000 ms and flip angle = 60°. For full data reduction and processing, see Burgy et al. 2016^9^.

Participants completed a passive picture-viewing task to index emotional reactivity and recovery (Supplementary Figure 2)^35^. The task consisted of 60 positive, negative, and neutral IAPS images (180 trials) divided over 5 blocks. A white fixation cross was displayed in the center of a black screen (1 s), followed by a picture (4 s). Participants were instructed to indicate the valence of each image via button press. Following picture offset, a second fixation screen was presented. Two thirds of trials included a neutral face condition (male face presented for 500 ms at 3 s post-picture offset). After the offset of face images, the fixation cross was represented with an inter-trial interval that varied from 5.5 to 17.6 s (M = 8.89 s), providing sufficient variation to estimate the evoked BOLD response function. The remaining trials had no face presentation. The presentation of the faces allowed a behavioral measure of post-image recovery. Faces following negative images were rated as less likable than faces following neutral images or novel faces^9^.

### Image processing and analysis

To examine reactivity and recovery effects, no face and neutral face trials were separated into two 6 s epochs beginning at IAPS image onset, with the first epoch representing the initial response to the image (reactivity), and the second representing the neural recovery to the image and response to the face presentation, where applicable and as we previously reported^9^. Thus, the recovery epoch in image + face trials are considered recovery *as* modified by a neutral stimulus presentation. Neural activity was quantified as the percent signal change (PSC) from baseline in each epoch. To unconfound recovery from reactivity, initial PSC in the reactivity epoch was regressed onto PSC estimates of modulated recovery. Face trials were calculated with a double-subtraction of face and no face trials: (Negative IAPS image with Face—Neutral IAPS image with Face)—(Negative IAPS image without Face—Neutral IAPS image without Face). These contrasts were warped to MNI space, smoothed (FWHM = 6 mm), and intrapair contrasts were calculated. Both voxelwise and amygdala seed data were examined (seeds were 4 mm spheres described above in rs-FC for both right and left amygdala).

### Library preparation and high-throughput sequencing of genomic DNA

Whole blood was collected from each participant in a BD vacutainer CPT cell preparation tube with sodium heparin (cat # 362753). The peripheral blood mononuclear cells were isolated and genomic DNA was extracted using Promega wizard genomic DNA purification kit (cat #A1120), following the manufacturer’s protocol.

Genome-wide methylation data were generated by WuXi NextCode for each sample using whole-genome sequencing technologies from Illumina (HiSeq X). Briefly, genomic DNA (200 ng) was randomly fragmented, end-repaired, and ligated to NEBNext Methylated Adapter for Illumina following the manufacturer’s protocol (Illumina). Adapter-ligated DNA fragments, ranging from 200 to 400 base pairs (bp), were purified by Sample Purification Beads (Illumina) and then treated with sodium bisulfite (ZymoResearch EZ DNA methylation gold kit), which converts unmethylated cytosines to uracil and leaves methylated cytosines unchanged. Libraries of converted DNA fragments were then amplified using KAPA HiFi Hot Start Uracil + Ready Mix (KAPA Biosystems KM2801), Index Primer for Illumina and Universal PCR Primer for Illumina (NEB E7336A), and amplicons were purified by Sample Purification Beads (Illumina) and sequenced on a Next-Generation sequencer (Illumina HiSeq X). This approach yielded 650–800 million 150 bp-reads for each library. Image processing and sequence extraction used the standard Illumina Pipeline.

### DNA methylation detection

Quality control, mapping, and extraction of methylation information from the whole genome sequence data were done using bowtie2^36^ and bismark (version 0.17.0)^37–39^. The average number of raw reads for each sample (*N* = 6) was 404 million reads giving an average genomic coverage of 20.23× (median genomic coverage 19.53×). The sequence reads were filtered for low quality and adaptor sequences were removed. The cleaned reads were then mapped to the human reference genome (hg38) and an average of 283.3 million uniquely mapped reads were obtained for each sample, giving an average coverage of 14.16× (median coverage 13.86×). Sequence reads from both DNA strands (forward and reverse) were combined to determine the DNA methylation level at all CpG dinucleotides (~25.3 million). Differentially methylated regions (DMRs) were identified using the DSS-single analysis method^40,41^, which was selected because it incorporates the read depth into the DMR analysis and relies on smoothing so that neighborhood CpGs can be viewed as pseudo-replicates and dispersion can be estimated across an entire genomic window. All default settings were used in the DSS package (including a smoothing span of 500 bp). Notably, sex chromosomes were excluded from DMR analysis because the samples included both male and female pairs. DMRs were identified using DSS, and limiting DMRs to those having a minimum of 5 CpG dinucleotides and a difference in mean methylation of 10% between the two groups.

### Gene ontologies

Gene Ontology (GO) analysis was conducted in R using the ‘topGO’ package. This package calculates the *P*-values for over-representation of a set of genes to specific Gene Ontology terms. It uses a hypergeometric test to calculate the P-values, given a set of genes and a gene universe (i.e., the super set from which the smaller gene set is drawn).

### Sequence motif discovery

To identify sequence motif enrichments, the center coordinate was first identified for each DMR, then extended ±500 bp around this center coordinate. Sequences were next obtained for these coordinates using the human genome (hg38) and placed in the Discriminative Regular Expression Motif Elicitation (DREME) tool to identify enriched short, ungapped motifs from these sequences relative to random sequences used as a background^42^. Finally, enriched motifs were placed in SpaMo to predict transcription factors that may putatively bind to these discovered motifs^43^.

## Results

### Familial comparisons for behavioral and biological measures

To characterize non-shared environmental influences on measures of human anxiety, including generalized anxiety disorder (GAD), social phobia, HPA activity (cortisol concentration), and amygdala function, we computed intraclass correlations (ICCs)) to quantify the degree of within-pair similarity between MZ cotwins over a seven-year period^44^. For MZ twins, a low ICC indicates that the trait has low heritability and large non-shared environmental influences. The ICCs for GAD and social phobia dropped substantially from a high of .56 at age 8 (*P*-value < .001; *N* = 299 pairs) to a low of .40 at age 15 (*P*-value < 0.001; *N* = 214 pairs; Supplementary Table 2). This reduction in twin pair similarity is consistent with an age-related increase in non-shared environmental influences that has been observed for social and biological phenotypes^45^.

Despite finding that afternoon cortisol concentrations at age 8 showed high intrapair similarity (ICC = .73, *P*-value < 0.001; *N* = 299 pairs), we previously showed that afternoon basal cortisol is strongly influenced by shared environment, and the remaining variance in this early cortisol measure can be attributed to non-shared environmental influences^28^.

Finally, while initial amygdala activation (first 6 s of IAPS presentation) at age 15 was moderately familial (ICC = 0.44, *P*-value = 0.05; *N* = 24 pairs), our measure of interest, amygdala *recovery*, showed low and statistically non-significant twin pair similarity (ICC = −0.19, *P*-value = 0.75; *N* = 24 pairs). These data suggest that the time course for recovery (i.e., reduced amygdala BOLD signal) is particularly sensitive to a non-shared environment (Supplementary Table 2). Together, the analyses of these three variables (anxiety symptoms, late afternoon basal cortisol, and amygdala BOLD recovery) indicate that both behavioral indicators and biological measures related to anxiety are influenced by non-shared environmental factors.

### Predictions of intrapair MRI and anxiety differences by intrapair cortisol differences

We next employed the MZ twin difference design to determine if intrapair differences in cortisol predict intrapair *differences* in anxious behaviors in the full MZ twin sample. For this analysis, we regressed intrapair differences in two anxiety measures (GAD and social phobia) at ages 13 and 15 years on intrapair differences in afternoon cortisol at age eight. The cortisol differences predicted co-twin differences in general anxiety at age 13 years (*β* = 0.18, *P*-value = .03; DF = 125; *R* ^2^ = .03; *N* = 127 pairs) and social phobia at age 15 years (*β* = 0.16, *P*-value = .05; DF = 149; *R* ^2^ = .02; *N* = 151 pairs). Together with our previous findings, that showed a positive relationship between intrapair differences in childhood afternoon cortisol levels and intrapair differences in protracted amygdala activation in adolescence^9^, these data suggest that the MZ difference design could be used to select uniquely discordant twins based on significant differences in these measures, which might provide an opportunity to examine possible environmentally sensitive molecular changes (i.e., epigenetic changes) related to twin discordance in biological systems related to anxiety.

**Fig. 1:**
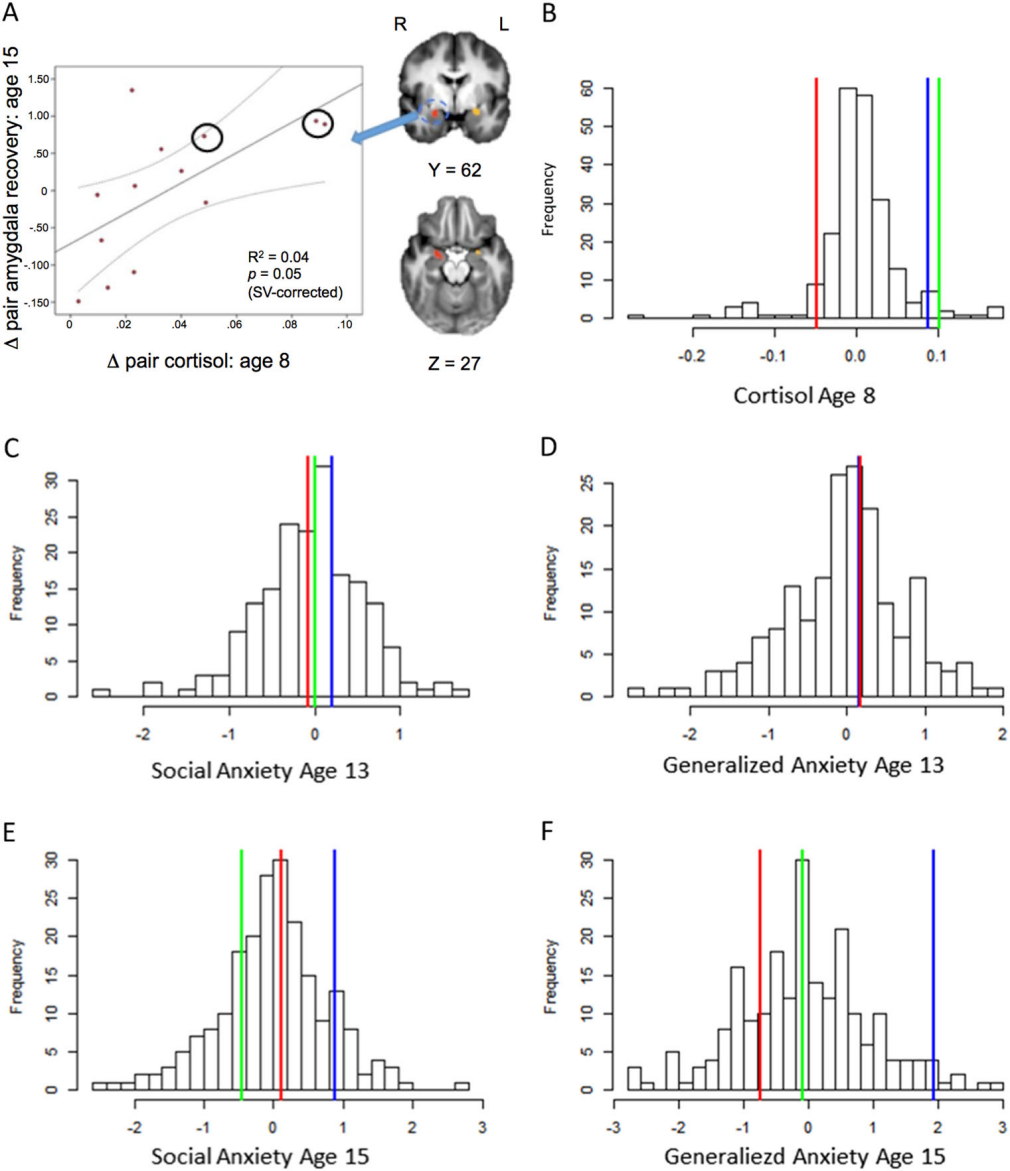
Intrapair differences in childhood afternoon cortisol, amygdala function and behavior **a** The correlations between the intrapair differences in childhood cortisol (*x* axis) and amygdala-modulated recovery (*y* axis) are shown. Note: Twin pairs selected for whole genome epigenetic analysis are circled in black. **b**–**f** The distribution of the twin differences for the full sample is shown for HPA activity (cortisol level; **b**; *N* = 299) and anxious behaviors (**c**–**f**; *N* = 184 to 215). The selected pairs are indicated by color showing their relative twin difference compared to the full sample (pair A = green; B = red; C = green)

Using this approach, we selected three twin pairs that were the most discordant along two axes—cortisol levels at age eight and amygdala-modulated recovery at age 15—for whole-genome analysis of DNA methylation. These time points were chosen because all three selected pairs were clearly discordant for HPA activity at age 8 years (Fig. 1b), but they did not become discordant for behavioral measures of anxiety until age 15 years (Figs. 1c–f). Interviews with a trained clinician during the neuroimaging visit (see Materials and Methods) confirmed that the more anxious twin had a current clinical diagnosis of a generalized anxiety or social phobia and the less anxious co-twin did not have a current clinical anxiety diagnosis. It should be noted that in all three pairs the less anxious co-twin had either a past disorder or past or current sub-threshold symptoms. Whole blood samples were obtained from the three twin pairs (2 female) 2–5 years after neuroimaging, as young adults (mean age = 20 years±1.2 years; Supplementary Table 1 **;** Fig. 1).

### Detection of anxiety-related DNA methylation in human blood

To identify susceptibility loci related to anxiety and its biological correlates, genomic DNA isolated from blood cells of each individual was treated with sodium bisulfite and whole-genome sequenced on a next-generation sequencer (see Materials and Methods). This approach generated an average of ~404 million raw sequence reads for each sample and, after filtering for quality, an average of 283.3 million sequence reads uniquely mapped to the human reference genome (hg38), giving an average genomic coverage of >14× (Supplementary Table 3). These methylation data were then restricted to only CpG dinucleotides that had high-quality data in all six samples (*N* = 23,444,312 CpG dinucleotides) and this final dataset revealed a bimodal distribution of DNA methylation in human blood cells, with the majority (>60%) of CpGs being more than 60% methylated (Supplementary Figure 3).

To examine whether blood harbors differential DNA methylation that is related to individual differences in anxiety, the methylation data were subjected to a differential methylation analysis that employed a statistical algorithm that incorporates sequence data read depth and does not need data from biological replicates (Materials and Methods). This analytical approach, which limited positive results to differentially methylated regions (DMRs) that have a minimum of 5 adjacent CpG dinucleotides and a minimum mean intrapair methylation difference of 10% across the three twin pairs, revealed a total of 230 anxiety-related differentially methylated loci. Anxiety-related increases in methylation were classified as hyper-DMRs and anxiety-related decreases in methylation were classified as hypo-DMRs. A total of 176 hyper- and 54 hypo-DMRs were identified and these loci were distributed across all the autosomes (Supplementary Figure 4; Dataset 1), suggesting a genome-wide increase in DNA methylation is associated with anxiety, a finding that is consistent with previous studies^25^.

**Fig. 2:**
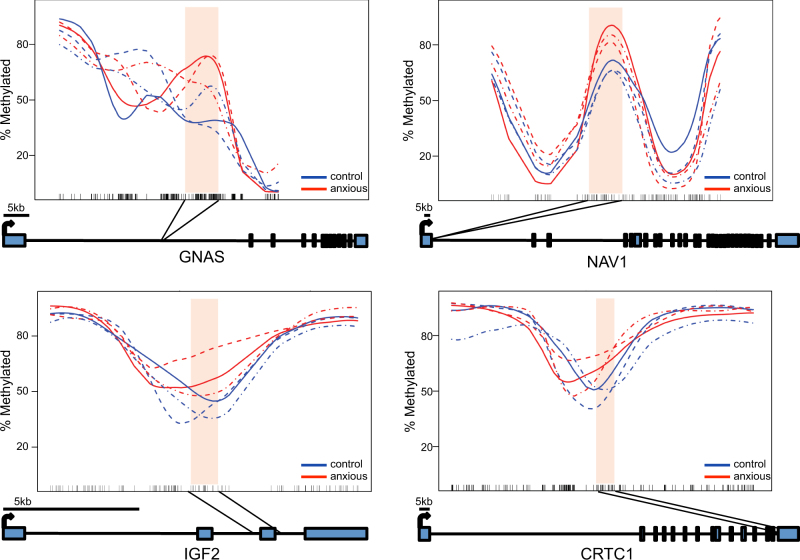
Representative regions of differential methylation in human anxiety A smoothing plot that shows the relative location of differentially methylated (*y* axis) CpG dinucleotides (*x* axis; black tick marks) in *GNAS* (**a**), *NAV1* (**b**), *IGF2* (**c**), and *CRTC1* (**d**). The DNA methylation profiles for the anxious (red) and unaffected (control; blue) twin-pairs are shown and the genomic region of significance between twin-pairs is highlighted (peach). Each corresponding co-twin is indicated by a different line pattern (pair A = solid; B = dashed; C = dash + dot)

Annotation of the anxiety-related DMRs to genes revealed 183 genes, many of which were known stress-related genes such as *NAV1*, *IGF2*, *GNAS*, and *CRTC1* (Fig. 2; Dataset 1). These data suggest that differential methylation in blood may reveal susceptibility loci in human anxiety. We next examined the gene ontologies (GOs) of these 183 anxiety-related genes and found several biologically relevant ontological terms that were among the differentially methylated genes, including regulation of dendrite development and neuron differentiation (Dataset 2). Many of the anxiety-related genes that contributed to these ontological terms have been previously implicated in psychiatric-related disorders, such as *CUX2*, *SYN3*, and *JAG2* ^46,47^. Together, these findings suggest that differential methylation levels in the blood are associated with relevant neurodevelopmental pathways that may contribute to the anxious phenotype.

As an initial validation of these findings, we tested for significance in an overlap with anxiety-related differentially methylated loci that we previously reported in the central nucleus of the amygdala of young monkeys^26^ and found a significant overlap (*P*-value < 0.05) of anxiety-related differentially methylated genes, including *GNAS*, *SYN3*, and *JAG2* (Table 1). Notably, three of these genes contained differential methylation of the exact same CpG dinucleotides in the human and monkey genomes (*GNAS*, *PLEC*, and *DOK3*). Together, these data suggest a commonality in the molecular underpinnings of anxiety in monkeys and humans, providing validation of our multi-species approach and promising candidate genes.

**Table 1:** Common anxiety-related differentially methylated genes between human and rhesus monkey

Gene Symbol	Sig.^*^	Chr	CpG#/Window size	Gene name
GNAS**	599.4	20	55/478	G-protein alpha subunit
*TRAPPC9*	228.3	8	30/437	Trafficking protein particle complex 9
*NFIC*	190.4	19	29/461	Nuclear factor 1 C-type
*MPL*	128.9	1	19/178	Myeloproliferative Leukemia
*JAG2*	109.0	14	18/338	Jagged-2
*GRB10*	101.7	7	15/102	Growth factor receptor-bound protein 10
*TET3*	87.9	2	15/233	Tet Methylcytosine Dioxygenase 3
*RGS12*	87.7	4	12/100	Regulator of G-protein signaling 12
*MAGI1*	87.0	3	12/214	Membrane-associated guanylate kinase
PLEC**	76.3	8	13/102	Plectin
*ZNF579*	48.7	19	8/160	Zinc Finger Protein 579
*KLF6*	38.6	10	7/111	Krueppel-like factor 6
*ZNF385A*	36.6	12	9/331	Zinc Finger Protein 385 A
*RYR1*	29.6	19	5/105	Ryanodine receptor 1
*ZBTB7A*	23.8	19	5/68	Zinc finger and BTB domain-containing protein 7 A
*SYN3*	−32.5	22	7/121	Synapsin-3
*MAP2K4*	−64.5	17	9/67	Dual specificity mitogen-activated protein kinase 4
DOK3**	−78.5	5	14/188	Docking protein 3

*Significance determined by DSS-single analysis method^39,40^

**Genes containing differential methylation of the same CpG dinucleotides in human and monkey

**Fig. 3:**
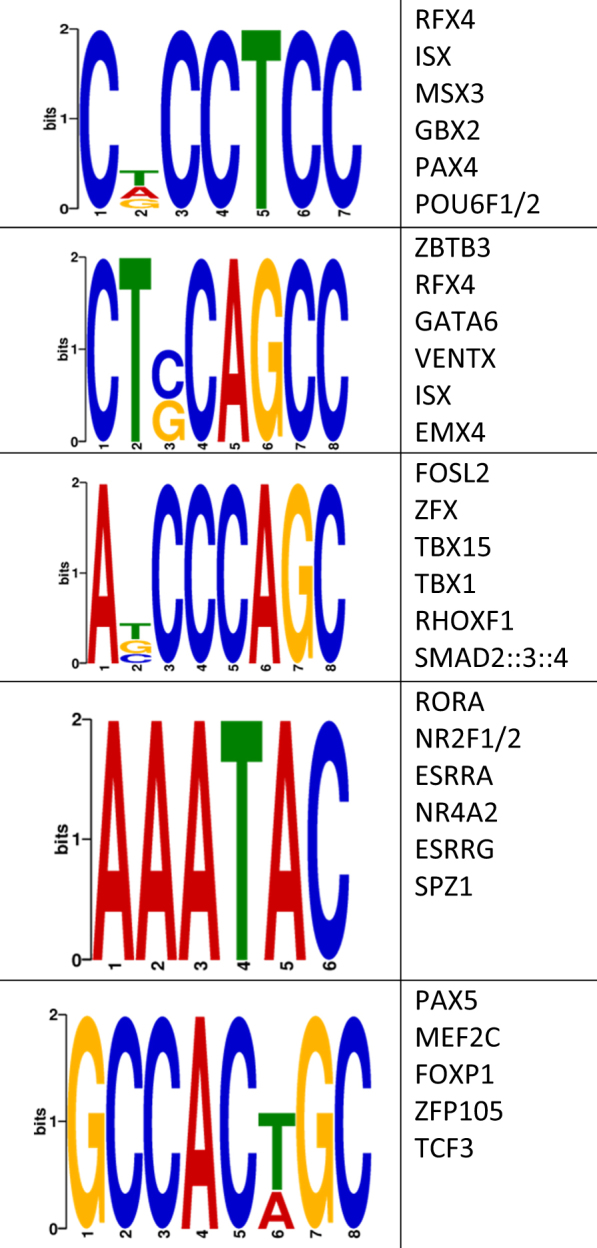
Characterization of a potential role(s) of DMRs in gene expression Identification of DMR-associated transcription factor sequence motifs that were predicted by the DREME suite (*E*-value < 10e−3). The putative transcription binding factors were predicted using SpaMo directly from the DREME suite and are shown next to each sequence motifs

Finally, we investigated whether the human anxiety-related DMRs contained enrichments of known transcription factor (TF) binding sequences, using the Discriminative Regular Expression Motif Elicitation (DREME) suite software package (Materials and Methods)^42^. This analysis yielded five TF binding sequences that were significantly enriched within the DMR sequences (Fig. 3). These TF binding sequences preferentially bind to several transcription factors, many of which have biologically relevant links, including the development of the central nervous system (*TCF3*) and general development (*RORA* and *SMAD2:3:4*; Fig. 3; see Discussion)^48–50^. Together, these data support previous reports^51–53^ of differential methylation modulating transcription factor binding, which here may alter gene expression related to the development of anxiety.

## Discussion

Many twin studies have investigated the extent to which anxiety is influenced by latent environmental factors^17^. Epigenetic modifications are one mechanism by which experiential factors influence anxious behaviors. Our goal was not to examine a representative sample, but to maximize MZ intrapair differences to determine the potential for epigenetic modifications in human blood related to human anxiety. We identified 230 loci with anxiety-related DNA methylation differences in blood of MZ twins that were selected based on discordance in cortisol and MRI measures related to anxiety. The power of whole genome sequence data was critical to detection, as mean methylation levels are strongly correlated across the genome and statistical power was increased by ‘borrowing’ strength across adjacent measurements^54^.

Although using relevant tissue is important for the biological interpretation of epigenome-wide association studies, obtaining such samples can be challenging for human studies of brain-related disorders. The significant overlap with the previously reported anxious temperament-associated differentially methylated loci from a key component of the neural circuit underlying primate anxiety provides a corroboration of the genes found here and suggests candidate genes, including *GNAS*, *SYN3*, and *JAG2*, for future mechanistic studies in the nonhuman primate model.

A cautionary note is that comparison of these genes to those from a meta-analysis of genome-wide association studies for anxiety disorders did not find an overlap of genes^55^. We might have expected some overlap under the hypothesis that sequence variants in the same genes could have similar biological effects to changes in DNA methylation. Nonetheless, several genes that we detected with DMRs have well-known links to neurodevelopmental disorders. For example, the stimulatory G-protein alpha subunit (*GNAS*) is located in a complex imprinted locus whose gene products are involved in early postnatal adaptations and neuroendocrine functions^56^. Maternal stress during pregnancy is linked with differential methylation on *GNAS* and insulin-like growth factor 2 (*IGF2*) gene^57^, which also was found here with an anxiety-related DMR. Synapsin 3 (*SYN3*) is a member of a gene family that encodes neuronal phosphoproteins that associate with the cytoplasmic surface of synaptic vesicles^47^. *SYN3* has a role in synaptogenesis and the modulation of neurotransmitter release, leading to its link to several neuropsychiatric conditions, including bipolar disorder, autism, schizophrenia and epilepsy^58,59^. We previously reported anxiety-related differential methylation of *JAG1* and *JAG2* in the central nucleus of the amygdala of young monkeys;^26^ here we find anxiety-related differential methylation of *JAG2* in humans. These genes are biologically relevant candidate genes contributing to anxiety because they are *NOTCH* receptor genes that play a critical role in brain development, adult synaptic plasticity, and memory formation. Finally, these findings also revealed potentially novel genes contributing to anxiety (e.g., *PLEC* and *DOK3*) and 87 DMRs that were not associated with any genes, suggesting that they may reside in non-coding regions of the genome and warrant a deeper investigation.

Several transcription factors that recognize significantly enriched sequence motifs within the DMRs have known roles in neurogenesis and neurological activities. Transcription factor 3 (*TCF3*) is a neurodevelopmental transcription factor that directly enhances the expression of *HES1*, which regulates the development of the central nervous system through *NOTCH* signaling^50^. The Retinoid-Related Orphan Receptor-Alpha (*RORA*) is a key regulator of embryonic development, cellular differentiation, immunity, and circadian rhythm^60–62^. *RORA* is linked with an increased risk for several neuropsychiatric conditions, including autism spectrum, bipolar disorder, schizophrenia, depression, and fear-related psychopathology^48,63,64^. Finally, the dysregulation of *SMAD* genes reportedly affect oligodendrogenesis^49^, axon development and regeneration, growth and maintenance of midbrain dopaminergic neurons^65^. Alterations in *SMAD*-related molecular cascades are reported for various psychiatric conditions^66–70^.

In addition, several of the genes that encode the transcription factors, such as *TCF3* and a member of the Kruppel-like factor (*KLF*) family (*KLF-6*), contain DMRs, suggesting that DNA methylation may also disrupt the expression of transcription factors. The involvement of DNA methylation in the regulation of KLF family members is important because they are required for late phase neuronal maturation in the developing dentate gyrus during adult hippocampal neurogenesis^71^. Murine studies find associations with *Klf-9* and anxiety, and human studies link *Klf-11* with chronic stress and depressive disorders^71,72^. While these data support previous reports^51–53,73–76^ of differential methylation associated with transcription factor binding sites, it is notable that here, and in these reports, the majority of transcription factor binding sites do not contain CpG dinucleotides directly in the transcription factor binding motif. Recent data suggest that TF complexes may contain DNA methyltransferases, implying that some TFs may function to bring DNA methyltransferases to genomic regions of interest^77^. Clearly, further functional studies are needed to definitively determine the role of differential methylation within transcription factor binding sites related to anxiety.

Although the twin pairs were clearly discordant for HPA activity at age 8 years, they did not become discordant for behavioral measures of anxiety until mid-adolescence. In fact, the selected co-twins, and the sample in general, are remarkably similar on anxiety measures at age 13 years, and start to diverge only 2 years later. This finding may not be surprising given the increased prevalence for anxious behaviors that coincide with the onset of puberty^78^. Nonetheless, sensitivity to daily stressors, as embodied by environmentally mediated variability in basal cortisol levels, may be an early, subtle indicator of vulnerability to later anxiety symptoms. Although the twin pairs were not selected on measures of anxiety, all three were discordant in the same direction such that the co-twin with higher cortisol and longer amygdala recovery was also the more anxious co-twin. At the individual level, HPA function at age 8 years was not related to anxiety longitudinally or concurrently in the full sample. The association between HPA function and anxious behavior was only revealed after controlling for unknown genetic and environmental confounds shared by twins reared together. Finally, it is notable that the selected twins reported fluctuations in the level of anxiety between the imaging and blood draw visits. While there are a number of reasons that a diagnosis could change between visits, including reluctance to disclose symptoms, effective treatment of prior symptoms, and spontaneous remediation, the changes in DNA methylation that are linked to adolescent anxiety status appears to be stable and may be useful for long-term diagnostics and treatment strategies.

It should be noted that the original anxiety-related changes in DNA methylation were found in young monkeys (<2 years) that had never received anxiety-related medications, which are commonly administered to humans with anxiety disorders. Together, these study design parameters improve the chances that the identified anxiety-related changes in DNA methylation have etiological relevance rather than being a consequence of treatment. Because none of the twin pairs were on any medications (aside from hormonal birth control; *N* = 1; Supplementary Table 1) and we found a significant overlap of anxiety-related changes in human blood, these data support that the changes found in humans may have etiological relevance.

An important strength of this study was the longitudinal multi-source data—cortisol levels at age eight and anxiety and amygdala BOLD signal measures at age 15 years—that were used for twin pair phenotyping and selection. Coupled with the power of whole genome sequencing, each step of this approach was critical for the detection of anxiety-related DNA methylation differences in human blood. Together, these findings underscore the value of using multiple and longitudinal biological and behavioral markers to identify novel peripheral molecular differences linked to experience-dependent anxiety in a monozygotic twin sample.

### Data access

We have submitted the data generated from the monkey methylation data for this study to the Gene Expression Omnibus (GEO), which can be found under the Gene Series: TBD.

## Electronic supplementary material

Supplemental Figure 1

Supplemental Figure 2

Supplemental Figure 3

Supplemental Figure 4

Supplemental Table 1

Supplemental Table 2

Supplemental Table 3

## Footnotes

1 MZ co-twins are identical for DNA sequence variants with the exception of rare somatic mutations. MZ twins reared together also share many non-genetic factors (e.g. age, parenting and so on); thus, reliable MZ twin* differences* are attributed to unique or non-shared environmental factors. In this context, “environmental” simply means “non-genetic” and “unique” means “not shared with the co-twin.”

## References

[1] Gross C, Hen R (2004). The developmental origins of anxiety. Nat. Rev. Neurosci..

[2] Kagan J, Snidman N (1999). Early childhood predictors of adult anxiety disorders. Biol. Psychiatry.

[3] Kalin NH, Shelton SE (2003). Nonhuman primate models to study anxiety, emotion regulation, and psychopathology. Ann. N. Y. Acad. Sci..

[4] Shirtcliff EA, Essex MJ (2008). Concurrent and longitudinal associations of basal and diurnal cortisol with mental health symptoms in early adolescence. Dev. Psychobiol..

[5] Schmidt LA (1997). Behavioral and neuroendocrine responses in shy children. Dev. Psychobiol..

[6] Klimes-Dougan B, Hastings PD, Granger DA, Usher BA, Zahn-Waxler C (2001). Adrenocortical activity in at-risk and normally developing adolescents: individual differences in salivary cortisol basal levels, diurnal variation, and responses to social challenges. Dev. Psychopathol..

[7] Essex MJ, Klein MH, Cho E, Kalin NH (2002). Maternal stress beginning in infancy may sensitize children to later stress exposure: effects on cortisol and behavior. Biol. Psychiatry.

[8] Burghy CA (2012). Developmental pathways to amygdala-prefrontal function and internalizing symptoms in adolescence. Nat. Neurosci..

[9] Burghy CA (2016). Experience-driven differences in childhood cortisol predict affect-relevant brain function and coping in adolescent monozygotic twins. Sci. Rep..

[10] Erickson K, Drevets W, Schulkin J (2003). Glucocorticoid regulation of diverse cognitive functions in normal and pathological emotional states. Neurosci. Biobehav. Rev..

[11] Gunnar M, Quevedo K (2007). The neurobiology of stress and development. Annu. Rev. Psychol..

[12] Urry HL (2006). Amygdala and ventromedial prefrontal cortex are inversely coupled during regulation of negative affect and predict the diurnal pattern of cortisol secretion among older adults. J. Neurosci..

[13] Bishop S, Duncan J, Brett M, Lawrence AD (2004). Prefrontal cortical function and anxiety: controlling attention to threat-related stimuli. Nat. Neurosci..

[14] Stein MB, Simmons AN, Feinstein JS, Paulus MP (2007). Increased amygdala and insula activation during emotion processing in anxiety-prone subjects. Am. J. Psychiatry..

[15] Phelps EA, LeDoux JE (2005). Contributions of the amygdala to emotion processing: from animal models to human behavior. Neuron..

[16] Kalin NH, Shelton SE, Davidson RJ (2004). The role of the central nucleus of the amygdala in mediating fear and anxiety in the primate. J. Neurosci..

[17] Shackman, A. J. et al. Heightened extended amygdala metabolism following threat characterizes the early phenotypic risk to develop anxiety-related psychopathology. *Mol. Psychiatry* **22**, 724–732 (2016). 10.1038/mp.2016.132 PMC5332536 27573879

[18] Hettema JM, Neale MC, Kendler KS (2001). A review and meta-analysis of the genetic epidemiology of anxiety disorders. Am. J. Psychiatry.

[19] Abdolmaleky HM (2006). Hypomethylation of MB-COMT promoter is a major risk factor for schizophrenia and bipolar disorder. Hum. Mol. Genet..

[20] Poulter MO (2008). GABAA receptor promoter hypermethylation in suicide brain: implications for the involvement of epigenetic processes. Biol. Psychiatry.

[21] Kuratomi G (2008). Aberrant DNA methylation associated with bipolar disorder identified from discordant monozygotic twins. Mol. Psychiatry.

[22] Collishaw S (2007). Resilience to adult psychopathology following childhood maltreatment: evidence from a community sample. Child Abuse Negl..

[23] Kappeler L, Meaney MJ (2010). Epigenetics and parental effects. Bioessays.

[24] Weaver IC (2004). Epigenetic programming by maternal behavior. Nat. Neurosci..

[25] Murphy TM (2015). Anxiety is associated with higher levels of global DNA methylation and altered expression of epigenetic and interleukin-6 genes. Psychiatr. Genet..

[26] Alisch RS (2014). Differentially methylated plasticity genes in the amygdala of young primates are linked to anxious temperament, an at risk phenotype for anxiety and depressive disorders. J. Neurosci..

[27] Bartels M, de Geus EJ, Kirschbaum C, Sluyter F, Boomsma DI (2003). Heritability of daytime cortisol levels in children. Behav. Genet..

[28] Van Hulle CA, Shirtcliff EA, Lemery-Chalfant K, Goldsmith HH (2012). Genetic and environmental influences on individual differences in cortisol level and circadian rhythm in middle childhood. Horm. Behav..

[29] Renteria ME (2014). Genetic architecture of subcortical brain regions: common and region-specific genetic contributions. Genes Brain Behav..

[30] Waszczuk MA, Zavos HM, Gregory AM, Eley TC (2014). The phenotypic and genetic structure of depression and anxiety disorder symptoms in childhood, adolescence, and young adulthood. JAMA Psychiatry..

[31] Schmidt NL (2013). Wisconsin Twin Research: early development, childhood psychopathology, autism, and sensory over-responsivity. Twin research and human genetics: the official journal of the International Society for Twin Studies.

[32] Shaffer D, Fisher P, Lucas CP, Dulcan MK, Schwab-Stone ME (2000). NIMH Diagnostic Interview Schedule for Children Version IV (NIMH DISC-IV): description, differences from previous versions, and reliability of some common diagnoses. J. Am. Acad. Child Adolesc. Psychiatry.

[33] Lucas CP (2001). The DISC Predictive Scales (DPS): efficiently screening for diagnoses. J. Am. Acad. Child. Adolesc. Psychiatry.

[34] Kaufman J (1997). Schedule for affective disorders and schizophrenia for school-age children-present and lifetime version (K-SADS-PL): initial reliability and validity data. J. Am. Acad. Child Adolesc. Psychiatry.

[35] Schuyler BS (2014). Temporal dynamics of emotional responding: amygdala recovery predicts emotional traits. Soc. Cogn. Affect. Neurosci..

[36] Langmead B, Salzberg SL (2012). Fast gapped-read alignment with Bowtie 2. Nat. Methods..

[37] Krueger F, Andrews SR (2011). Bismark: a flexible aligner and methylation caller for Bisulfite-Seq applications. Bioinformatics.

[38] Tsuji J, Weng Z (2016). Evaluation of preprocessing, mapping and postprocessing algorithms for analyzing whole genome bisulfite sequencing data. Brief Bioinform..

[39] Kunde-Ramamoorthy G (2014). Comparison and quantitative verification of mapping algorithms for whole-genome bisulfite sequencing. Nucleic Acids Res..

[40] Wu H (2015). Detection of differentially methylated regions from whole-genome bisulfite sequencing data without replicates. Nucleic Acids Res..

[41] Feng H, Conneely KN, Wu H (2014). A Bayesian hierarchical model to detect differentially methylated loci from single nucleotide resolution sequencing data. Nucleic Acids Res..

[42] Bailey TL (2011). DREME: motif discovery in transcription factor ChIP-seq data. Bioinformatics.

[43] Whitington T, Frith MC, Johnson J, Bailey TL (2011). Inferring transcription factor complexes from ChIP-seq data. Nucleic Acids Res..

[44] McGraw KO, Gordji S, Wong SP (1994). How many subjects to screen? A practical procedure for estimating multivariate normal probabilities for correlated variables. J. Consult. Clin. Psychol..

[45] Bergen SE, Gardner CO, Kendler KS (2007). Age-related changes in heritability of behavioral phenotypes over adolescence and young adulthood: a meta-analysis. Twin Res Hum Genet.

[46] Gonzalez-Mantilla AJ, Moreno-De-Luca A, Ledbetter DH, Martin CL (2016). A cross-disorder method to identify novel candidate genes for developmental brain disorders. JAMA Psychiatry.

[47] Kao HT (1998). A third member of the synapsin gene family. Proc Natl Acad Sci USA..

[48] Hamilton BA (1996). Disruption of the nuclear hormone receptor RORalpha in staggerer mice. Nature..

[49] Nicolay DJ, Doucette JR, Nazarali AJ (2007). Transcriptional control of oligodendrogenesis. Glia..

[50] Ikawa T, Kawamoto H, Goldrath AW, Murre C (2006). E proteins and Notch signaling cooperate to promote T cell lineage specification and commitment. J. Exp. Med..

[51] Li S (2016). Genome-wide alterations in hippocampal 5-hydroxymethylcytosine links plasticity genes to acute stress. Neurobiol. Dis..

[52] Papale LA (2016). Sex-specific hippocampal 5-hydroxymethylcytosine is disrupted in response to acute stress. Neurobiol. Dis..

[53] Papale, L. A., Madrid, A., Li, S., Alisch, R. S. Early-life stress links 5-hydroxymethylcytosine to anxiety-related behaviors. *Epigenetics*. **12**, 264–276 (2017). 10.1080/15592294.2017.1285986 PMC5398765 28128679

[54] Michels KB (2013). Recommendations for the design and analysis of epigenome-wide association studies. Nat. Methods..

[55] Otowa T (2016). Meta-analysis of genome-wide association studies of anxiety disorders. Mol. Psychiatry..

[56] Williamson CM (2004). A cis-acting control region is required exclusively for the tissue-specific imprinting of Gnas. Nat. Genet..

[57] Vangeel EB (2015). DNA methylation in imprinted genes IGF2 and GNASXL is associated with prenatal maternal stress. Genes Brain Behav..

[58] Cruceanu C (2013). H3K4 tri-methylation in synapsin genes leads to different expression patterns in bipolar disorder and major depression. Int. J. Neuropsychopharmacol..

[59] Griswold AJ (2012). Evaluation of copy number variations reveals novel candidate genes in autism spectrum disorder-associated pathways. Hum. Mol. Genet..

[60] Chen XR (2013). Mature Purkinje cells require the retinoic acid-related orphan receptor-alpha (RORalpha) to maintain climbing fiber mono-innervation and other adult characteristics. J. Neurosci..

[61] Dussault I, Fawcett D, Matthyssen A, Bader JA, Giguere V (1998). Orphan nuclear receptor ROR alpha-deficient mice display the cerebellar defects of staggerer. Mech. Dev..

[62] Boukhtouche F (2006). RORalpha, a pivotal nuclear receptor for Purkinje neuron survival and differentiation: from development to ageing. Cerebellum.

[63] Devanna P, Vernes SC (2014). A direct molecular link between the autism candidate gene RORa and the schizophrenia candidate MIR137. Sci. Rep..

[64] Miller MW, Wolf EJ, Logue MW, Baldwin CT (2013). The retinoid-related orphan receptor alpha (RORA) gene and fear-related psychopathology. J. Affect. Disord..

[65] Hegarty SV (2014). Canonical BMP-Smad signalling promotes neurite growth in rat midbrain dopaminergic neurons. Neuromol. Med..

[66] Toyooka K, Usui M, Washiyama K, Kumanishi T, Takahashi Y (2002). Gene expression profiles in the brain from phencyclidine-treated mouse by using DNA microarray. Ann. N. Y. Acad. Sci..

[67] Beech RD (2012). Altered expression of cytokine signaling pathway genes in peripheral blood cells of alcohol dependent subjects: preliminary findings. Alcohol. Clin. Exp. Res..

[68] Beech RD (2010). Increased peripheral blood expression of electron transport chain genes in bipolar depression. Bipol. Disord..

[69] Levey DF (2016). Towards understanding and predicting suicidality in women: biomarkers and clinical risk assessment. Mol. Psychiatry.

[70] Zaharieva I (2008). Association study in the 5q31-32 linkage region for schizophrenia using pooled DNA genotyping. BMC Psychiatry.

[71] Scobie KN (2009). Kruppel-like factor 9 is necessary for late-phase neuronal maturation in the developing dentate gyrus and during adult hippocampal neurogenesis. J. Neurosci..

[72] Duncan J, Johnson S, Ou XM (2012). Monoamine oxidases in major depressive disorder and alcoholism. Drug Discov. Ther..

[73] Domcke S (2015). Competition between DNA methylation and transcription factors determines binding of NRF1. Nature..

[74] Douet V, Heller MB, Le Saux O (2007). DNA methylation and Sp1 binding determine the tissue-specific transcriptional activity of the mouse Abcc6 promoter. Biochem. Biophys. Res. Commun..

[75] Murphy DM (2009). Global MYCN transcription factor binding analysis in neuroblastoma reveals association with distinct E-box motifs and regions of DNA hypermethylation. PLoS ONE.

[76] Tian HP (2015). DNA Methylation Affects the SP1-regulated Transcription of FOXF2 in Breast Cancer Cells. J. Biol. Chem..

[77] Yang R (2015). Hydrogen sulfide promotes Tet1- and Tet2-mediated Foxp3 demethylation to drive regulatory T cell differentiation and maintain immune homeostasis. Immunity..

[78] Reardon LE, Leen-Feldner EW, Hayward C (2009). A critical review of the empirical literature on the relation between anxiety and puberty. Clin. Psychol. Rev..

