# Supplementary figures and images for: A multi-dimensional characterization of anxiety in monozygotic twin pairs reveals susceptibility loci in humans
Source: Transl Psychiatry. 2017 Dec 11;7:1282. doi: 10.1038/s41398-017-0047-9 (PMC5802687; doi:10.1038/s41398-017-0047-9)

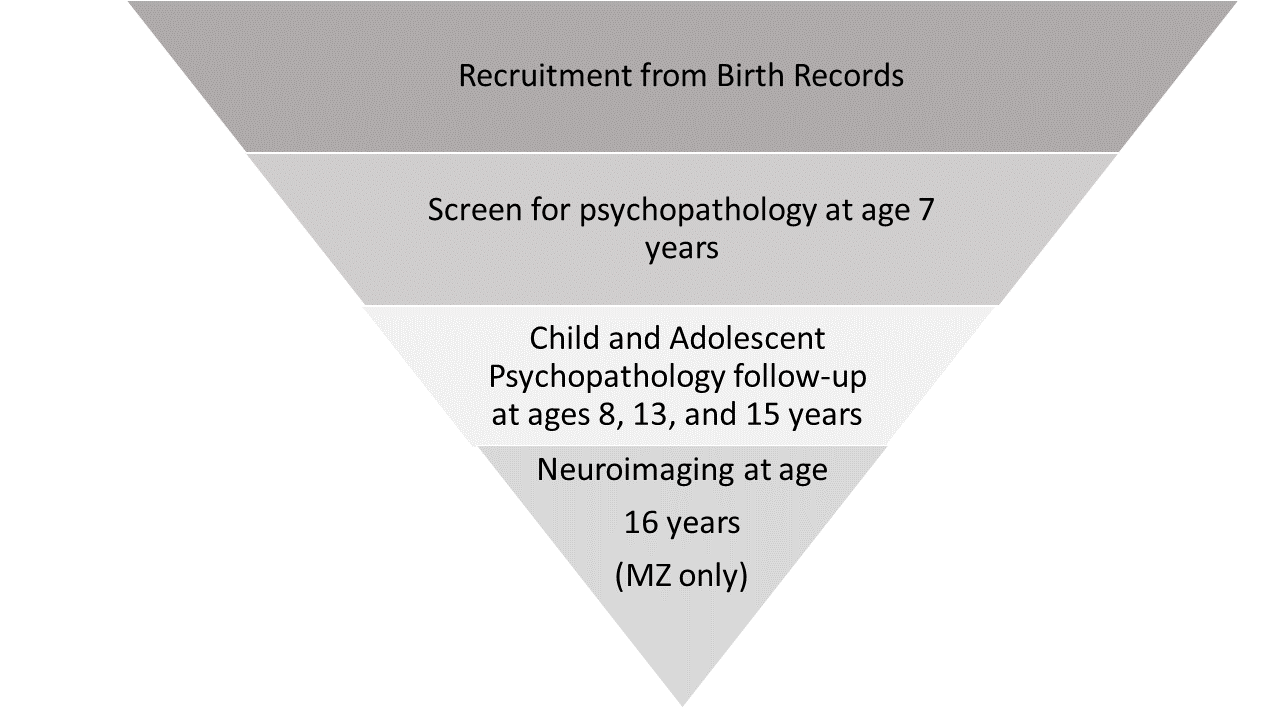

Supplement: Supplementary file 1 — Supplemental Figure 1 [file 41398_2017_47_MOESM1_ESM.docx]
