# Supplementary material for: A multi-dimensional characterization of anxiety in monozygotic twin pairs reveals susceptibility loci in humans
Source: Transl Psychiatry. 2017 Dec 11;7:1282. doi: 10.1038/s41398-017-0047-9 (PMC5802687; doi:10.1038/s41398-017-0047-9)
Supplement: Supplementary file 5 — Supplemental Table 1 [file 41398_2017_47_MOESM5_ESM.docx]

Supplementary Table 1: Description of full twin sample and three selected twin pairs

|  | Full Sample*  *N* = 299 pairs | pair A | pair B | pair C |
| --- | --- | --- | --- | --- |
| *Demographics* |  |  |  |  |
| Gender (% female) | 50.3 | F | M | F |
| ethnicity (% white) | 93 | White | Hispanic | White |
| Chorionicity | ⎯ | Di | Di | Mono |
| Mother’s Education (years) | 15.3 | 12 | 12 | 20 |
| Income (in thousand) | $50-$60 | $50-$60 | $80-$90 | $50-$60 |
| *Age in years* |  |  |  |  |
| Wave 1 | 7.9 (0.8) | 8.8 | 8.6 | 7.3 |
| Wave 2 | 13.5 (1.6) | 15 | 14 | 12 |
| Wave 3 | 15.1 (1.9) | 16 | 16 | 13 |
| Imaging visit^a^ | 15.8 (1.6) | 18 | 18 | 13 |
| Blood draw | ⎯ | 22 | 22 | 18 |
| *Behavior* |  |  |  |  |
| Chronic Anxiety  Pair Discordance | 13.5%  5% | Both  Concordant | One  Neither | One  Discordant |
| Medication use | ⎯ | One^b^ | None | None |
| Current Anxiety Disorder^c^ | ⎯ | Both | None | None |

^*^ Excluding DZ twin pairs

^a^ Total imaging sample consists of 25 twin pairs, 58% female; selected pair A & B had puberty status 5 (fully mature), pair C had puberty status 3.5 at the imaging visit.

^b^ High anxious twin reported using hormonal birth control at time of blood draw.

^c^ At time of blood draw.

Note. Demographics taken from wave 1 assessment; chorionicity is derived from birth records. Participants were considered to have chronic anxiety if they met diagnostic interview criteria for an anxiety disorder at two waves (parent or self-report). Participants were considered non-anxious if they did not meet diagnostic interview criteria at any wave (parent and self-report). Pairs were discordant if one twin was chronically anxious and his/her co-twin was non-anxious; pairs were concordant if both twins were chronically anxious.
