# Supplementary material for: A multi-dimensional characterization of anxiety in monozygotic twin pairs reveals susceptibility loci in humans
Source: Transl Psychiatry. 2017 Dec 11;7:1282. doi: 10.1038/s41398-017-0047-9 (PMC5802687; doi:10.1038/s41398-017-0047-9)
Supplement: Supplementary file 6 — Supplemental Table 2 [file 41398_2017_47_MOESM6_ESM.docx]

Supplementary Table 2: Intraclass correlations for MZ twins.

|  | Age 8  (*n* = 299) | Age 13  (*n* = 184) | Age 15  (*n* = 214) | Age 16  (*n* = 13^a^) |
| --- | --- | --- | --- | --- |
| Cortisol | .73 |  |  |  |
| GAD Composite | .55 | .56 | .41 |  |
| Social Phobia | .56 | .56 | .40 |  |
| Amygdala activation^a^ |  |  |  | .44 |
| Amygdala recovery^a^ |  |  |  | -.19 |

^a^ imaged pairs without age 8 cortisol were excluded from analyses. Missing cortisol was unrelated to study variables.

Note. All correlations are significant at p<.01, except Amygdala recovery. Age 8 anxiety composites are based on parent reports; Age 13 and 15 anxiety composites are based on adolescent self-report; GAD = generalized anxiety disorder composite; Amygdala recovery = activity during the recovery period regressed on amygdala activity during the reactivity period[^10^](#_ENREF_10).
