# Supplementary material for: A multi-dimensional characterization of anxiety in monozygotic twin pairs reveals susceptibility loci in humans
Source: Transl Psychiatry. 2017 Dec 11;7:1282. doi: 10.1038/s41398-017-0047-9 (PMC5802687; doi:10.1038/s41398-017-0047-9)
Supplement: Supplementary file 7 — Supplemental Table 3 [file 41398_2017_47_MOESM7_ESM.docx]

| Supplementary Table 3: Summary of Sequence Data | |  |
| --- | --- | --- |
| **Sample ID** | **Number of raw Seq reads** | **Number of unique alignments** |
| WGC078008F | 391708186 | 275866366 |
| WGC078009F | 373295483 | 265147604 |
| WGC078010F | 383563506 | 270299962 |
| WGC078011F | 389789234 | 278697314 |
| WGC101306F | 444324336 | 298442824 |
| WGC101307F | 444948529 | 311155850 |
| Average | 404604879 | 283268320 |
| Genomic Coverage (ave.) | 20.23024395 | 14.163416 |
