# Supplementary material for: A multi-dimensional characterization of anxiety in monozygotic twin pairs reveals susceptibility loci in humans
Source: Transl Psychiatry. 2017 Dec 11;7:1282. doi: 10.1038/s41398-017-0047-9 (PMC5802687; doi:10.1038/s41398-017-0047-9)
Supplement: Supplementary file 6 [file 41398_2017_47_MOESM2_ESM.docx]

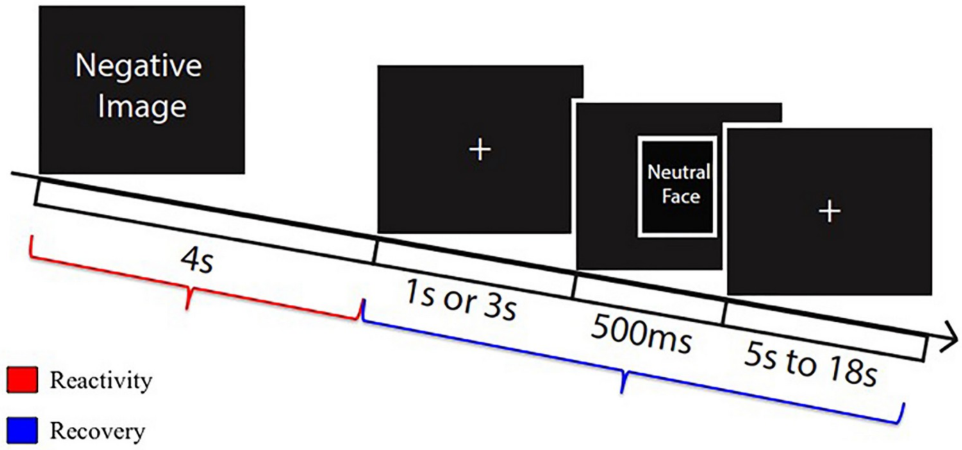
Negative Image
+
Neutral Face
+
4s
1s or 3s
500ms
5s to 18s
Reactivity
Recovery
